# Use of Patient-Reported Outcome Measures and Patient-Reported Experience Measures Within Evaluation Studies of Telemedicine Applications: Systematic Review

**DOI:** 10.2196/30042

**Published:** 2021-11-17

**Authors:** Andreas Knapp, Lorenz Harst, Stefan Hager, Jochen Schmitt, Madlen Scheibe

**Affiliations:** 1 Center for Evidence-Based Healthcare University Hospital Carl Gustav Carus, Carl Gustav Carus Faculty of Medicine Technische Universität Dresden Dresden Germany; 2 Comprehensive Pain Center University Hospital Carl Gustav Carus Dresden Dresden Germany

**Keywords:** telemedicine, telehealth, evaluation, outcome, patient-reported outcome measures, patient-reported outcome, patient-reported experience measures, patient-reported experience, measurement instrument, questionnaire

## Abstract

**Background:**

With the rise of digital health technologies and telemedicine, the need for evidence-based evaluation is growing. Patient-reported outcome measures (PROMs) and patient-reported experience measures (PREMs) are recommended as an essential part of the evaluation of telemedicine. For the first time, a systematic review has been conducted to investigate the use of PROMs and PREMs in the evaluation studies of telemedicine covering all application types and medical purposes.

**Objective:**

This study investigates the following research questions: in which scenarios are PROMs and PREMs collected for evaluation purposes, which PROM and PREM outcome domains have been covered and how often, which outcome measurement instruments have been used and how often, does the selection and quantity of PROMs and PREMs differ between study types and application types, and has the use of PROMs and PREMs changed over time.

**Methods:**

We conducted a systematic literature search of the MEDLINE and Embase databases and included studies published from inception until April 2, 2020. We included studies evaluating telemedicine with patients as the main users; these studies reported PROMs and PREMs within randomized controlled trials, controlled trials, noncontrolled trials, and feasibility trials in English and German.

**Results:**

Of the identified 2671 studies, 303 (11.34%) were included; of the 303 studies, 67 (22.1%) were feasibility studies, 70 (23.1%) were noncontrolled trials, 20 (6.6%) were controlled trials, and 146 (48.2%) were randomized controlled trials. Health-related quality of life (n=310; mean 1.02, SD 1.05), emotional function (n=244; mean 0.81, SD 1.18), and adherence (n=103; mean 0.34, SD 0.53) were the most frequently assessed outcome domains. Self-developed PROMs were used in 21.4% (65/303) of the studies, and self-developed PREMs were used in 22.3% (68/303). PROMs (n=884) were assessed more frequently than PREMs (n=234). As the evidence level of the studies increased, the number of PROMs also increased (τ=−0.45), and the number of PREMs decreased (τ=0.35). Since 2000, not only has the number of studies using PROMs and PREMs increased, but the level of evidence and the number of outcome measurement instruments used have also increased, with the number of PREMs permanently remaining at a lower level.

**Conclusions:**

There have been increasingly more studies, particularly high-evidence studies, which use PROMs and PREMs to evaluate telemedicine. PROMs have been used more frequently than PREMs. With the increasing maturity stage of telemedicine applications and higher evidence level, the use of PROMs increased in line with the recommendations of evaluation guidelines. Health-related quality of life and emotional function were measured in almost all the studies. Simultaneously, health literacy as a precondition for using the application adequately, alongside proper training and guidance, has rarely been reported. Further efforts should be pursued to standardize PROM and PREM collection in evaluation studies of telemedicine.

## Introduction

### Background

With the rise of digital health technologies and telemedicine services, the need for evidence-based evaluation is growing [1]. Over the past years, several evaluation guidelines that address study types, outcomes, and patient perspectives, among other requirements have been published [2-7]. The two best-known and most commonly used evaluation guidelines are the *Model for Assessment of Telemedicine* (MAST) *applications* [2] and the *evidence standards framework for digital health technologies* of the English National Institute for Health and Care Excellence (NICE framework) [3]. They have been used in several evaluation studies over the years [1,8-10].

Focusing on outcomes, MAST provides the following elements as part of a multidisciplinary evaluation of telemedicine applications: clinical effectiveness, patient perspective, safety, economic aspects, organizational aspects, and sociocultural, ethical, and legal aspects [2]. The patient’s perspective is evaluated by patient-reported outcome measures (PROMs), such as health-related quality of life (HRQoL) or behavioral outcomes, the latter being relevant when focusing on the domain of clinical effectiveness. In addition, patient-reported experience measures (PREMs) should be a part of the evaluation to assess satisfaction and acceptance, understanding of information, confidence in the treatment, ability to use the application, and empowerment [2]. The NICE framework provides minimum evidence standards and best practice standards for the evaluation of digital health technologies according to the degree of the treatment. Among them are, for example, the demonstration of effectiveness, use of behavior change techniques, and economic aspects. It also recommends the assessment of patient-centered outcomes in complex digital health technologies and specifically states that many of these outcomes should be measured using PROMs [3]. This demonstrates the importance of PROM and PREM in the context of evaluation studies of telemedicine applications.

The US Food and Drug Administration refers to PROMs as “any reports coming directly from patients about how they function or feel in relation to a health condition and its therapy, without interpretation of the patient’s responses by a clinician, or anyone else” [11]. These reports are ideally collected using validated outcome measurement instruments (OMIs), which are regarded as cost-effective, efficient, and scalable, especially in the early stages of development of an innovative intervention [1]. In addition, PROMs are classified according to generic, disease-specific, and target group–specific OMIs [12].

OMIs that quantify the experience, satisfaction, acceptance, or quality of care from the patients’ perspective are called PREMs. The goal of PREMs is to measure and report whether the provided care meets the expectations of the patients. Thus, PREMs are an indicator of patient centeredness and service quality in health care [13].

In the past, PROMs and PREMs have been used to evaluate the effectiveness and quality of care achieved when implementing telemedicine applications. Reviews of evaluation studies regarding telemedicine applications showed that single outcome domains such as HRQoL and psychological outcomes were used for specific use cases, such as inflammatory bowel disease management [14], adherence, self-efficacy, and self-management for medication management [15]. PREMs were used, for example, to measure satisfaction with knee pain management [16].

In summary, PROMs and PREMs have been recommended and already used for the evaluation of telemedicine applications. However, to the best of our knowledge, no systematic review exists to date that investigates the characteristics of the use of PROMs and PREMs in evaluation studies of telemedicine applications irrespective of application type and medical purpose.

It is still not known which and how often outcome domains and OMIs have been used in evaluation studies and whether the selection and frequency differ by the characteristics of the telemedicine application and the chosen study type. Our systematic review was conducted to close this research gap.

### Objectives

This review aims to investigate the following research questions:

1. In which scenarios have PROMs and PREMs been collected for evaluation purposes?
2. Which PROM and PREM outcome domains have been covered and how often?
3. Which OMIs have been used and how often?
4. Did the selection and quantity of PROMs and PREMs differ between study types and application types?
5. Has the use of PROMs and PREMs in evaluation studies changed over time?

Furthermore, we will assess the extent to which the results can be transferred to use cases that have been derived from frequent combinations of application types and medical purposes.

## Methods

### Systematic Literature Research

To identify relevant articles, we conducted an electronic database search on MEDLINE and Embase. On the basis of the Population, Intervention, Comparison, Outcome, Studies scheme, the following inclusion and exclusion criteria were defined (Textbox 1):

**Inclusion and exclusion criteria.**

**Patients**

- Inclusion criteria
  All patient groups with an indication for telemedicine care
- Exclusion criteria
  No patient group using telemedicine

**Intervention**

- Inclusion criteria
  Telemedicine applications with patients as main users
- Exclusion criteria
  Telemedicine applications with no patients as main users, for example, telecommunication between health professionals
  Telemedicine services containing a single telephone call or electronic message
  Telemedicine intervention addresses more than one International Statistical Classification of Diseases and Related Health Problems, 10th revision chapter (however, multiple conditions allowed within one International Statistical Classification of Diseases and Related Health Problems, 10th revision chapter); no telemedicine

**Control**

- Inclusion criteria
  Nontelemedical standard care (treatment as usual) or prospective designs
- Exclusion criteria
  Telemedicine versus telemedicine

**Outcome**

- Inclusion criteria
  Patient-reported outcome measures or patient-reported experience measures
- Exclusion criteria
  No patient-reported outcome measures or patient-reported experience measures

**Studies**

- Inclusion criteria
  Feasibility studies, noncontrolled trials, controlled trials, and randomized controlled trials
  Publications in English or German language
  No limitations on the date of publication
- Exclusion criteria
  Papers about telemedicine in general, guidelines and handbooks
  Reviews
  Case reports
  Retrospective studies
  Qualitative studies
  No English or German language

The search string (Multimedia Appendix 1) was based on 2 previous studies. The part dealing with the assessment of telemedicine applications is based on a review by Arnold and Scheibe et al [4], which aimed to identify standards for the evaluation of telemedicine applications. The part of the search string covering PROMs and PREMs is based on the PROM Group Construct and Instrument Type Filters of the University of Oxford [17]. This search string has already proven itself in the design of other reviews [18,19]. The search query was performed on April 2, 2020.

### Development of Data Extraction Matrix and Used Taxonomies

A matrix was developed as the basis for data extraction. The studies were categorized by (1) study type (feasibility study, noncontrolled trial, controlled trial, and randomized controlled trial [RCT]), (2) medical purpose (first letter of International Statistical Classification of Diseases and Related Health Problems, 10th revision [ICD-10] classification [20]), and (3) application type based on the taxonomy developed by Harst et al [21,22]. This taxonomy was chosen because of its development based on empirical data, which allows its use in quantifying and statistically analyzing the characteristics of telemedicine applications. This taxonomy differentiates between 6 different application types: (1) teleconsultation, a process of providing health care from health care providers to patients over a distance [23]; (2) telediagnostics, a process where a disease is identified over a distance [24]; (3) teleambulance or tele-emergency, a process where emergency care is assisted or data are collected during an emergency over a distance [25]; (4) telemonitoring, a process of data collection over a distance for the purpose of medical decision-making [23,26,27]; (5) telerehabilitation, a process of data collection over a distance for the purpose of coping with the long-term consequences of a disease or an impairment [28]; and (6) digital self-management, a process to promote responsibility for one’s own health and to encourage health literacy [29,30]. The classification into application types is intended to be the basis for subsequent subgroup analyses and has already been proven useful for this purpose in other systematic reviews evaluating telemedicine interventions [31,32].

All studies have been reviewed for the use of PROMs and PREMs; both could be represented by established and potentially validated OMIs, which were used frequently in nontelemedicine trials, or OMIs developed especially for the study in question. The OMIs were checked to verify whether they were established instruments or had been developed specifically for a study (SELF_PROM and SELF_PREM). The availability of a validation study served as an indicator of an established instrument. The psychometric properties of the OMIs were irrelevant for the classification into established and self-developed measures, as assessing the quality of the instrument was not within the scope of the review. The assignment of the OMIs to the individual outcome domains took place in an iterative process. In the first step, paraphrases were freely assigned to the OMIs. In the second step, the paraphrases were collected, mapped, and the corresponding categories were developed by the reviewers (AK and SH). The preliminary work of the Core Outcome Measures in Effectiveness Trials initiative provided the framework for the development of categories [33] but was supplemented by additional domains or modified where required. This was necessary, as the Core Outcome Measures in Effectiveness Trials initiative’s taxonomy does not sufficiently describe and categorize PREMs to fit the purpose of this review; thus, they had to be developed inductively from the collected and mapped paraphrases. Furthermore, categories were assigned to either the PROM or PREM areas. In the third step, OMIs were assigned to the previously defined outcome domains. To ensure objectivity in the assignment of outcome domains, the reviewers wrote a codebook in advance (Table 1).

**Table 1:** Codebook of the outcome domains.

Domain			Description^a^
**PROM^b^**			
	HRQoL^c^	Measures the HRQoL of the respondent	
	Physical function	Measures the extent to which the illness affects the physical function of the respondent	
	Social function	Measures the extent to which the illness affects the social function of the respondent	
	Emotional function	Measures the extent to which the illness affects the emotional function of the respondent	
	Cognitive function	Measures the extent to which the illness affects the cognitive function and disease perception of the respondent	
	Health literacy	Measures the respondent’s ability to avoid, alleviate, or live with a disease	
	Side effects	Measures complaints caused by therapeutic measures	
	Adherence	Measures the active role of the patient in the implementation of a therapy	
**PREM^d^**			
	Treatment	Deals with the experience of the medical component of a telemedical intervention	
	Technology	Deals with the experience of the technical component of a telemedical intervention	
	Satisfaction	Measures the general or overarching satisfaction with the telemedical intervention; satisfaction does not specifically target the medical or technical components of a telemedical intervention	

^a^The domain contains outcome measurement instruments.

^b^PROM: patient-reported outcome measure.

^c^HRQoL: health-related quality of life.

^d^PREM: patient-reported experience measure.

### Data Extraction

The developed matrix provided the basis for subsequent data extraction. The extraction of paper characteristics and information concerning study type, medical purpose, and application type was performed by 1 reviewer (AK) because of the limited risk of misinterpretation. A total of 2 reviewers (AK and SH) independently performed the assignment of OMIs to PROM and PREM outcome domains based on the developed codebook. In case of any disagreement, assignments were discussed and resolved by consent. The complete data extraction matrix can be found in Multimedia Appendix 1.

### Statistical Analysis

For the descriptive analysis, absolute and relative frequencies, mean values, and SDs were calculated for the individual outcome domains and for PROMs and PREMs. The calculations were performed once for all included studies as a whole and also individually for all study and application types. Correlation analyses according to Pearson for metric data and Kendall tau-b for ordinal data were performed to check the strength of dependencies.

To examine the transfer of results to individual subgroups, 3 use cases were selected from frequent combinations of medical purpose and application types. For this purpose, the frequent outcome domains and study types were determined and descriptively compared with the overall results.

## Results

### Study Selection

**Figure 1:**
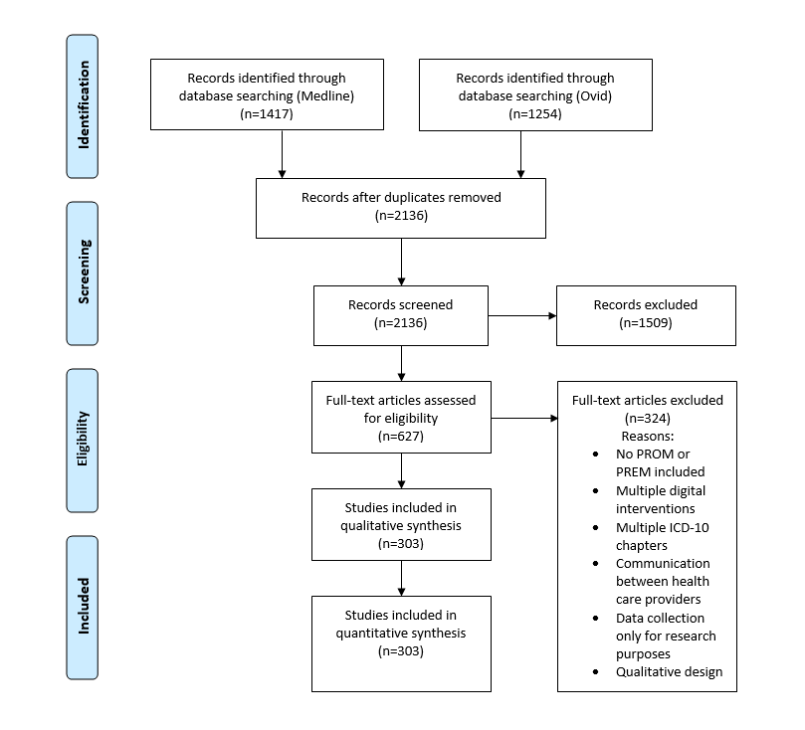
PRISMA (Preferred Reporting Items for Systematic Reviews and Meta-Analyses) flow chart. ICD-10: International Statistical Classification of Diseases and Related Health Problems, 10th revision; PREM: patient-reported experience measure; PROM: patient-reported outcome measure.

Overall, the electronic search resulted in 2671 hits. Of the 2671 studies, 2136 (79.97%) studies were included in the title abstract screening after removing duplicates. A total of 2 reviewers (AK and LH) performed this step. AK screened all the papers, and LH screened a sample to validate AK’s screening. The match between the reviewers was 82.3%, which, according to the AMSTAR 2 (A Measurement Tool to Assess Systematic Reviews) guidelines [34], legitimizes the examination of only a sample by a second reviewer. Of the 2136 papers, 627 (29.35%) papers were selected for full-text screening, which could be conducted by 1 reviewer (AK) because of the strictly formulated inclusion and exclusion criteria. Of the 627 papers, 303 (48.3%) papers were included in the review (Figure 1). A complete list of all inclusions can be found in Multimedia Appendix 2.

### Telemedicine Scenarios

All included studies (n=303) were categorized according to their medical purpose in terms of the ICD-10 chapter and the telemedicine application type (Table 2). The most common ICD-10 chapters were *I* for diseases of the circulatory system (51/303, 16.8%), *C* for neoplasm (47/303, 15.5%), and *F* for mental and behavioral disorders (44/303, 14.5%). Studies that could not clearly be assigned to a chapter were summarized under the term *other* (40/303, 13.2%). These studies were usually telemedicine applications from the fields of *primary prevention*, *aging*, and *well-being*.

**Table 2:** Identified scenarios of telemedicine applications evaluated via patient-reported outcome measures and patient-reported experience measures.

Application type		Teleambulance (N=0), n	Telediagnostics (N=4), n	Digital self-management (N=78), n	Teleconsultation (N=75), n	Telemonitoring (N=96), n	Telerehabilitation (N=50), n
**ICD-10^a^ chapter**							
	A^b^ (N=1), n	0	0	1	0	0	0
	B^b^ (N=9), n	0	0	4	3	2	0
	C^c^ (N=47), n	0	0	11	11	21	4
	D^c,d^ (N=0), n	0	0	0	0	0	0
	E^e^ (N=24), n	0	0	9	10	4	1
	F^f^ (N=44), n	0	1	13	22	6	2
	G^g^ (N=15), n	0	0	3	5	4	3
	H^h^ (N=3), n	0	1	0	2	0	0
	I^i^ (N=51), n	0	1	5	2	22	21
	J^j^ (N=19), n	0	0	3	2	9	5
	K^k^ (N=12), n	0	0	8	0	4	0
	L^l^ (N=6), n	0	1	3	3	1	0
	M^m^ (N=17), n	0	0	2	1	7	7
	N^n^ (N=6), n	0	0	2	3	1	0
	O^o^ (N=1), n	0	0	0	0	0	1
	P^p^ (N=0), n	0	0	0	0	0	0
	Q^q^ (N=2), n	0	0	1	0	1	0
	R^r^ (N=0), n	0	0	0	0	0	0
	S^s^ (N=2), n	0	0	0	0	0	2
	T^t^ (N=2), n	0	0	0	0	1	1
	V^u^ (N=0), n	0	0	0	0	0	0
	Z^v^ (N=0), n	0	0	0	0	0	0
	Other (N=40), n	0	0	14	10	13	3

^a^ICD-10: International Statistical Classification of Diseases and Related Health Problems, 10th revision.

^b^A-B: certain infectious and parasitic diseases.

^c^C-D: neoplasms.

^d^D: diseases of the blood and blood-forming organs and certain disorders involving the immune mechanism.

^e^E: endocrine, nutritional, and metabolic diseases.

^f^F: mental, behavioral, and neurodevelopmental disorders.

^g^G: diseases of the nervous system.

^h^H: diseases of the eye and adnexa; diseases of the ear and mastoid process.

^i^I: diseases of the circulatory system.

^j^J: diseases of the respiratory system.

^k^K: diseases of the digestive system.

^l^L: diseases of the skin and subcutaneous tissue.

^m^M: diseases of the musculoskeletal system and connective tissue.

^n^N: diseases of the genitourinary system.

^o^O: pregnancy, childbirth, and the puerperium.

^p^P: certain conditions originating in the perinatal period.

^q^Q: congenital malformations, deformations, and chromosomal abnormalities.

^r^R: symptoms, signs, and abnormal clinical and laboratory findings, not elsewhere classified.

^s^S: injury, poisoning, and certain other consequences of external causes.

^t^T: injury, poisoning, and certain other consequences of external causes.

^u^V: external causes of morbidity.

^v^Z: factors influencing health status and contact with health services.

Telemonitoring (96/303, 31.7%) was the most frequent type of application, followed by digital self-management (79/303, 26.1%), teleconsultation (75/303, 24.8%), and telerehabilitation (50/303, 16.5%), telediagnostics (4/303, 1.3%); there were no studies with teleambulance (0/303, 0%). The most common combinations of medical purpose and application type were diseases of the circulatory system+telemonitoring (22/303, 7.3%), mental and behavioral disorders+teleconsultation (22/303, 7.3%), diseases of the circulatory system+telerehabilitation (21/303, 6.9%), and neoplasm+telemonitoring (21/303, 6.9%). All other combinations were found in <20 cases. Of the 144 possible combinations, only 51 (35.4%) were identified in this study.

### Use of Outcome Domains

**Figure 2:**
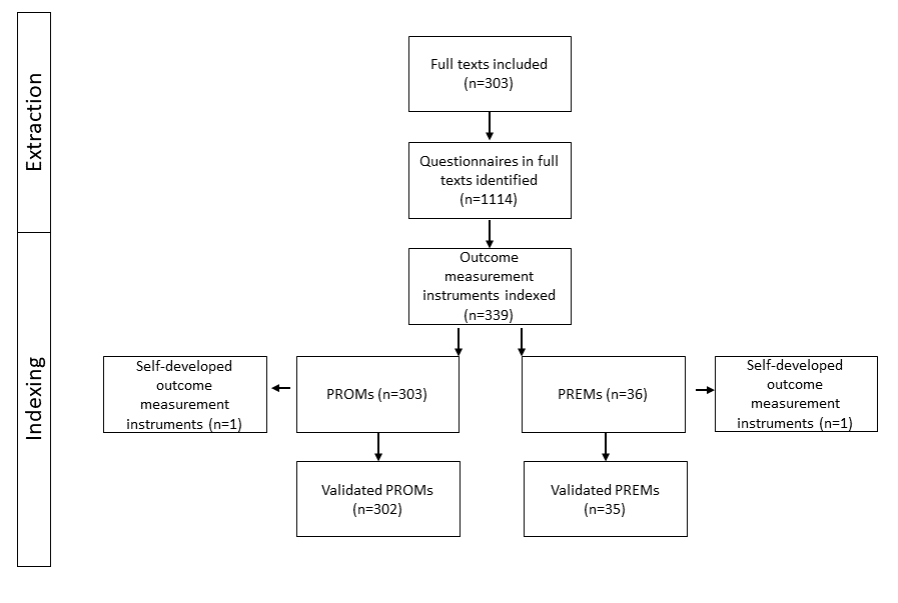
Extraction process of outcome measurement instruments. PREM: patient-reported experience measure; PROM: patient-reported outcome measure.

In total, 339 different OMIs were used in 1114 cases in the included studies (n=303). The OMIs were classified into 89.4% (303/339) PROMs and 10.6% (36/339) PREMs (Figure 2). Measurement instruments, which were developed especially for the individual study and were not listed in databases for PROMs and PREMs, were summarized in SELF_PROM or SELF_PREM. Measurement instruments for general satisfaction with the entire medical treatment process were summed up under the term SAT for satisfaction, which belongs to the field of PREMs and includes various forms of Likert scales, visual analog scales, and other self-developed constructs.

**Figure 3:**
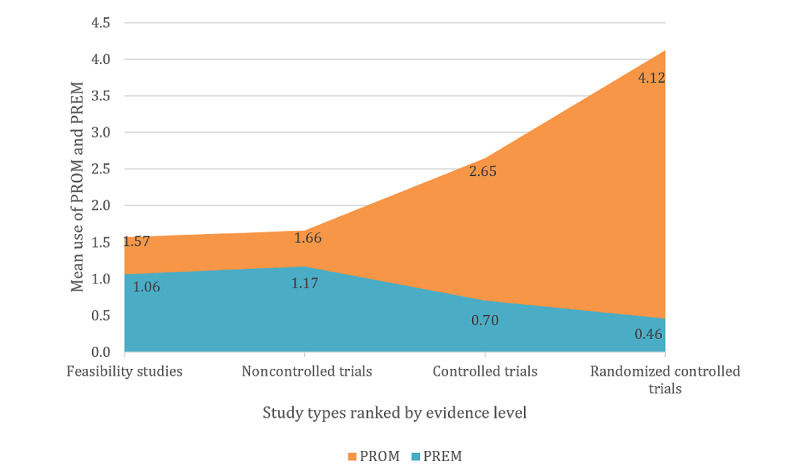
Use of patient-reported outcome measures and patient-reported experience measures by study type. PREM: patient-reported experience measure; PROM: patient-reported outcome measure.

Considering all studies, PROMs (881/1114, 79.08%) were used more frequently than PREMs (233/1114, 20.92%). The correlation analysis indicated that with an increasing number of PROMs, the number of PREMs decreased (r=−0.23; Figure 3). Across all studies, 21.4% (64/303) of PROMs and 22.3% (68/303) of PREMs were self-developed. The frequency of PROMs used was as follows (in descending order): HRQoL (310/881, 35.2%), emotional function (244/881, 27.7%), adherence (103/881, 11.7%), SELF_PROM (77/881, 8.7%), physical function (57/881, 6.5%), cognitive function (38/881, 4.3%), health literacy (35/881, 4%), social function (9/881, 1%), and side effects (8/881, 0.9%). The frequency of PREMs used was as follows (in descending order): general satisfaction (98/233, 42.1%), SELF_PREM (84/233, 36.1%), treatment (29/233, 12.4%), and technology (22/233, 9.4%).

**Figure 4:**
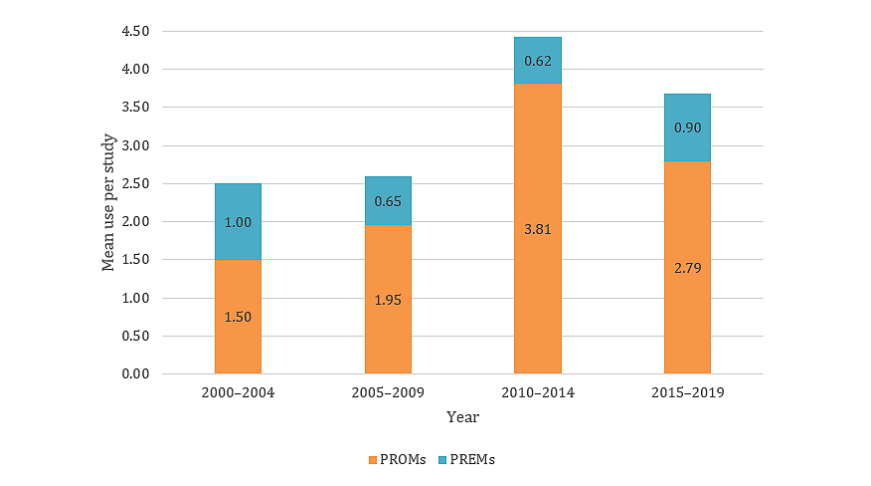
Mean use of patient-reported outcome measures and patient-reported experience measures over time. PREM: patient-reported experience measure; PROM: patient-reported outcome measure.

Considering the number of collected OMIs per study, it became apparent that most studies used 2-3 OMIs. The maximum number of OMIs used per study was 13 (Figure 4). Most OMIs used were PROMs (used in 881/1114, 79.08% of the included studies). In 15.5% (47/303) of the studies, no PROMs were used. The maximum was 11 PROMs per study (3/303, 1%). No PREMs were collected in 45.9% (139/303) of the studies. In 38.6% (117/303) of studies, one PREM was collected per study. The number declined sharply to 10.6% (32/303) of studies in which 2 PREMs were collected and fell further to the maximum of 0.3% (1/303) of studies in which 6 PREMs were collected.

### Outcome Measurement Instruments

The most commonly used PROM OMIs were the HRQoL OMIs EuroQol five-dimension scale [35] in 14.5% (44/303) of studies, the Short Form 36 [36] in 11.9% (36/303) of studies, and emotional function, especially depression symptoms, measured by the Patient Health Questionnaire-9 [37] in 8.9% (27/303) of studies.

The PREM OMIs that were most commonly used were the Client Satisfaction Questionnaire-8 to measure treatment satisfaction [38] in 4% (12/303) of studies and the System Usability Scale usability OMIs in the domain technology [39] in 2% (6/303) of studies. The third most frequently used OMI was the Patient Assessment of Chronic Illness Care OMI, which also measures treatment satisfaction [40], in 1% (3/303) of studies, together with the Telehealth Acceptance Measure [41], used in 1% (3/303) of studies.

The 3 most frequently used OMIs per outcome domain are listed in Table 3.

**Table 3:** Most frequently used outcome measurement instrument per outcome domain.

Outcome measurement instrument				Studies in the domain, N		Absolute frequency (n) and share in the domain, n (%)		Share in all studies (N=303), n (%)
**PROM^a^**								
	**HRQoL^b^**							
		EuroQol five-dimension scale	307		44 (14.3)		44 (14.5)	
		Short Form 36	307		36 (11.7)		36 (11.9)	
		Short Form 12	307		19 (6.2)		19 (6.3)	
	**Physical function**							
		International Physical Activity Questionnaire	57		7 (12.3)		7 (0.2)	
		Nottingham Extended Activities of Daily Living Scale	57		4 (7)		4 (1.3)	
		Active Australia Survey, Activities-specific Balance Scale, and Physical Activity Scale for the Elderly	57		3 (5.2)		3 (1)	
	**Social function**							
		Work Productivity and Activity Impairment Questionnaire	9		2 (22.2)		2 (0.7)	
		CHAMPS Activities Questionnaire for Older Adults, World Health Organization Health and Work Performance Questionnaire, Social Phobia Screening Questionnaire, and others	9		1 (11.1)		1 (0.3)	
	**Emotional function**							
		Patient Health Questionnaire-9	244		27 (11.1)		27 (8.9)	
		Hospital Anxiety and Depression Scale	244		23 (9.4)		23 (7.6)	
		Center for Epidemiologic Studies Depression Scale	244		16 (6.6)		16 (5.3)	
	**Cognitive function**							
		Brief Illness Perception Questionnaire	41		3 (7.3)		3 (1)	
		Supportive Care Needs Survey Short Form 34, Supportive Care Needs Survey Screening Tool 9, and Illness Perception Questionnaire	41		2 (4.9)		2 (0.7)	
		Body Attitude Test, Functional Activities Questionnaire, Illness Cognition Questionnaire, and others	41		1 (2.4)		1 (0.3)	
	**Health literacy**							
		European Heart Failure Self-Care Behaviour Scale and Self-Care of Heart Failure Index	35		3 (8.6)		3 (1)	
		Health Education Impact Questionnaire, Health Promoting Lifestyle Profile II, Patient Enablement Instrument, and others	35		2 (5.7)		2 (0.7)	
		Cancer Empowerment Questionnaire, Diabetes Self-Management Profile, Revised Heart Failure Compliance, and others	35		1 (2.9)		1 (0.3)	
	**Side effects**							
		Patient Neurotoxicity Questionnaire, Glasgow Antipsychotic Side-Effect Scale, Side Effects of Anti-epileptic Drugs, and others	8		1 (12.5)		1 (0.3)	
	**Adherence**							
		Morisky Medication Adherence Scale	103		5 (4.9)		5 (1.7)	
		Medication Adherence Rating Scale	103		4 (3.9)		4 (1.3)	
		AIDS Clinical Trails Group Adherence Questionnaire	103		1 (1)		1 (0.3)	
**PREM^c^**								
	**Treatment**							
		Client Satisfaction Questionnaire	29		12 (41.4)		12 (4)	
		Diabetes Treatment Satisfaction Questionnaire, Patient Assessment of Chronic Illness Care, and Patient Satisfaction Questionnaire Short Form	29		2 (6.9)		(0.7)	
		Canadian Health Care Evaluation Project questionnaire, Patient Experience Questionnaire, Functional Assessment of Chronic Illness Therapy–Treatment Satisfaction–Patient Satisfaction, and others	29		1 (3.4)		1 (0.3)	
	**Technology**							
		System Usability Scale	22		6 (27.3)		6 (2)	
		Telehealth Acceptance Measure	22		3 (13.6)		3 (1)	
		Post-Study System Usability Questionnaire and Usefulness, Satisfaction, and Ease of use Questionnaire	22		2 (12.3)		2 (0.7)	

^a^PROM: patient-reported outcome measure.

^b^HRQoL: health-related quality of life.

^c^PREM: patient-reported experience measure.

On average, each OMI was used 3.29 times, compared across all studies; however, most OMIs were only used once (modal value=1). There was a large variation in the frequency of use (SD 8.45) of single OMIs. Considering the frequency of use of single OMIs within the respective outcome domains, even the most frequently used OMIs, only achieved shares of ≤20% in the respective domains in most cases. This indicates a high heterogeneity of PROMs and PREMs used in the single outcome domains. To show this in a more differentiated manner, Table 4 indicates the absolute number of non–self-developed OMIs per outcome domain and their absolute frequency of use.

**Table 4:** Outcome measurement instrument per outcome domain in absolute numbers.

Outcome domain			Outcome measurement instruments (N=337), n (%)		Absolute frequency of uses (N=953), n (%)
**PROM^a^**					
	HRQoL^b^	109 (32.3)		310 (32.5)	
	Physical function	35 (10.4)		57 (6)	
	Social function	8 (2.4)		9 (0.9)	
	Emotional function	92 (27.3)		244 (25.6)	
	Cognitive function	28 (8.3)		38 (4)	
	Health literacy	16 (4.7)		35 (3.7)	
	Side effects	8 (2.4)		8 (0.8)	
	Adherence	6 (1.8)		103 (10.8)	
**PREM^c^**					
	Treatment	14 (4.2)		29 (3)	
	Technology	14 (4.2)		22 (2.3)	

^a^PROM: patient-reported outcome measure.

^b^HRQoL: health-related quality of life.

^c^PREM: patient-reported experience measure.

OMIs that were developed explicitly for use in telemedicine applications could only be identified for PREMs. These 6 OMIs were the Telehealth Acceptance Measure (3/233, 1.3%) [41], Mobile Application Rating Scale (1/233, 0.4%) [42], Patient Assessment of Communication during Telehealth (1/233, 0.4%) [43], Service User Technology Acceptability Questionnaire (1/233, 0.4%) [44], Telemedicine Perception Questionnaire (1/233, 0.4%) [45], and Telehealth Usability Questionnaire (1/233, 0.4%) [46]. Telemedicine-specific questionnaires were used in only 3.4% (8/233) of all PREMs.

### Chronological Trends in the Use of PROMs and PREMs

**Figure 5:**
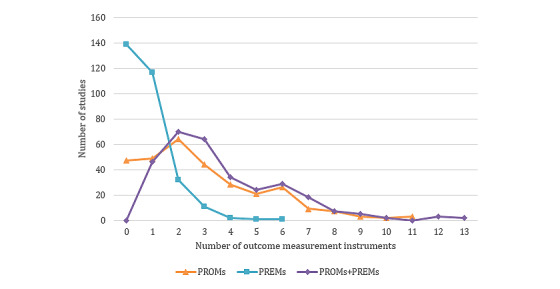
Number of outcome measurement instruments collected per study. PREM: patient-reported experience measure; PROM: patient-reported outcome measure.

The included studies were clustered into 5-year groups for analysis of the evaluation practice development over time (Figure 5). The year 2020 was not included in the analysis, as data were only available for the first 4 months of that year. The number of included studies increased above average over the years. The share of RCTs doubled every 5 years until 2014 and then dropped from 68.5% (50/73) to 43.7% (73/166) from 2014 to 2019.

**Figure 6:**
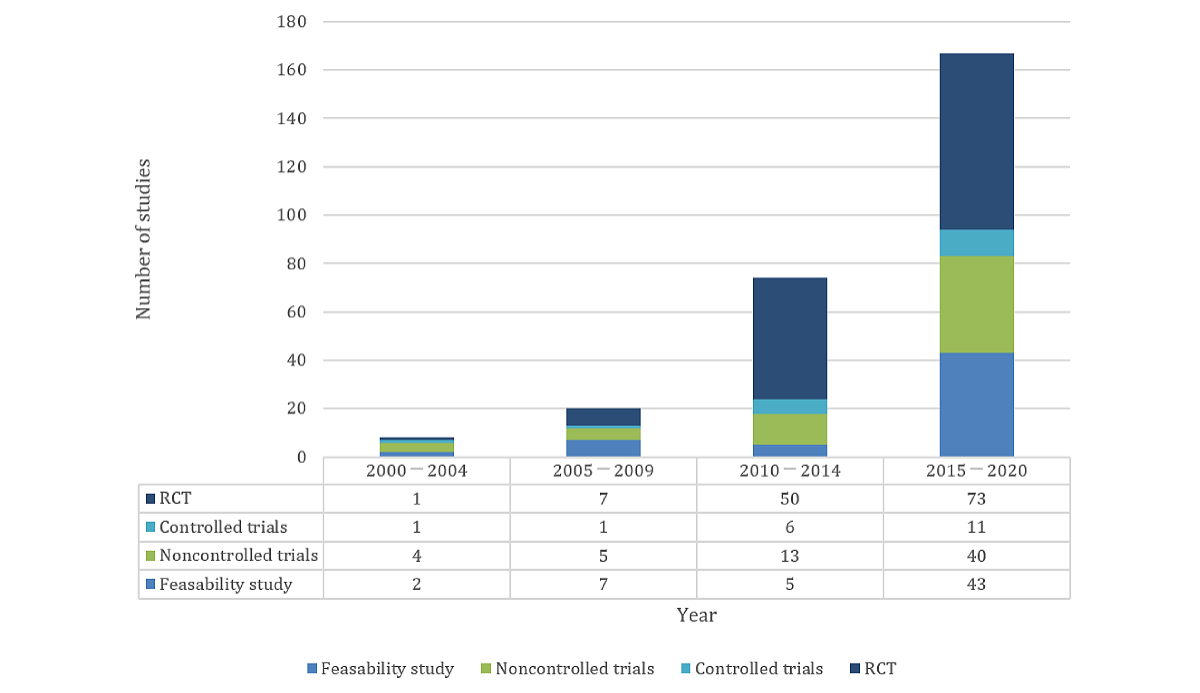
Numbers of studies by study type over time. RCT: randomized controlled trial.

The average use of PROMs per study, as well as the total number of OMIs used, steadily increased between 2000 and 2014 and then decreased between 2015 and 2019 (Figure 6). The mean use of PREMs per study remained at a lower level permanently compared with PROMs.

**Figure 7:**
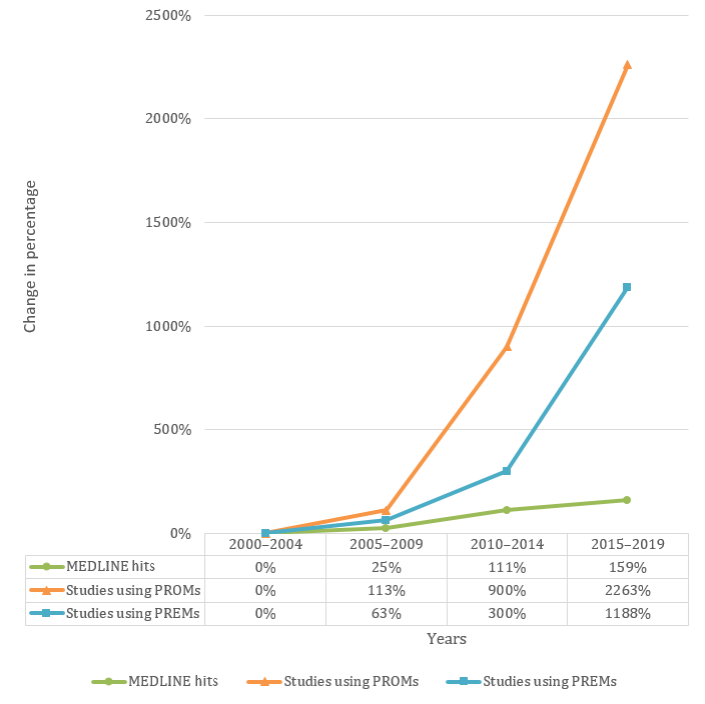
Change over time. PREM: patient-reported experience measure; PROM: patient-reported outcome measure.

To examine the change in the evaluation of telemedicine over time, the 2000-2004 episode was used as a starting point (Figure 7). The percentage increase or decrease compared with that in 2000-2004 was calculated. In addition, the number of telemedicine studies, regardless of whether they used a single PROM and PREM, was determined by hits of the term *telemedicine* in MEDLINE per year. These were compared with the included studies that used PROMs and PREMs for evaluation.

The number of telemedicine studies has steadily increased over time. However, the number of studies reporting PROMs and the number of studies reporting PREMs increased more compared with MEDLINE hits.

### Subgroup Analysis: Application Type

**Figure 8:**
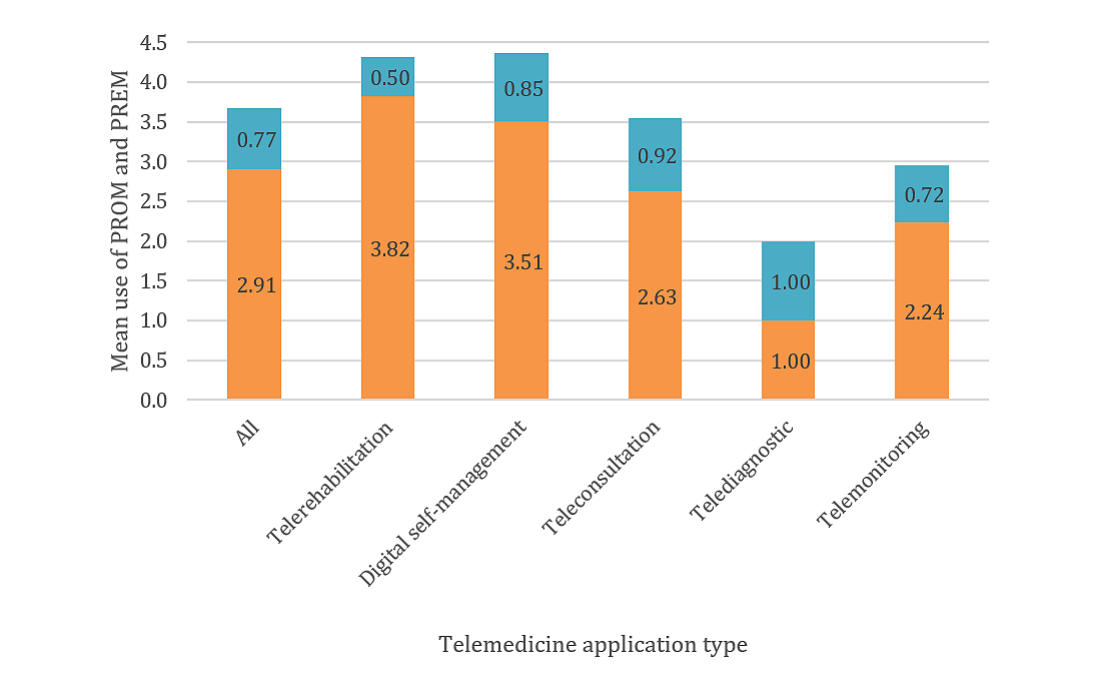
Use of patient-reported outcome measures and patient-reported experience measures by application type. PREM: patient-reported experience measure; PROM: patient-reported outcome measure.

Subgroup analysis for application type was conducted to cluster the technologies described in the studies according to their intended medical purpose and to explore differences in the evaluation approaches. On average, more PROMs were applied in studies focusing on telerehabilitation (mean 3.82, SD 2.60) and digital self-management (mean 3.51, SD 2.51) than on teleconsultation (mean 2.63, SD 2.41), telemonitoring (mean 2.24, SD 1.92), and telediagnostics (mean 1.00, SD 2.00). The application of PREMs was distributed evenly across all application types (range of mean values 0.50-1.06). Figure 8 shows the mean values of the PROMs and PREMs used by application type and compared with the mean values of all studies. The values for all the application types and outcome domains can be found in Table 5.

**Table 5:** Outcomes by application type (N=303).

Outcomes		All			Telediagnostics (n=4)			Digital self-management (n=78)			Teleconsultation (n=75)			Telemonitoring (n=96)			Telerehabilitation (n=50)	
		∑	Mean value (SD)	∑		Mean value (SD)	∑		Mean value (SD)	∑		Mean value (SD)	∑		Mean value (SD)	∑		Mean value (SD)
**PROM^a^**																		
	PROM (total)	884	2.91 (2.40)	4		1.00 (2.00)	274		3.51 (2.49)	197		2.63 (2.41)	215		2.24 (1.92)	191		1.40 (2.60)
	HRQoL^b^	310	1.02 (1.05)	1		0.25 (0.50)	78		1.00 (0.50)	65		0.87 (1.00)	94		0.98 (0.99)	72		1.44 (1.05)
	Physical function	57	0.19 (0.45)	0		0.00 (0.00)	17		0.22 (0.47)	6		0.08 (0.27)	8		0.08 (0.28)	26		0.52 (0.71)
	Social function	9	0.03 (0.17)	0		0.00 (0.00)	5		0.06 (0.25)	3		0.04 (0.20)	0		0.00 (0.00)	1		0.02 (0.14)
	Emotional function	244	0.80 (1.18)	2		0.50 (1.00)	82		1.05 (1.32)	75		1.00 (1.48)	42		0.44 (0.81)	43		0.86 (0.90)
	Cognitive function	38	0.13 (0.40)	0		0.00 (0.00)	13		0.17 (0.37)	9		0.12 (0.37)	6		0.06 (0.28)	10		0.20 (0.61)
	Health literacy	37	0.12 (0.38)	1		0.25 (0.50)	12		0.15 (0.45)	4		0.05 (0.28)	14		0.15 (0.43)	4		0.06 (0.27)
	Side effects	8	0.03 (0.18)	0		0.00 (0.00)	4		0.05 (0.27)	0		0.00 (0.00)	2		0.02 (0.14)	2		0.04 (0.20)
	Adherence	103	0.34 (0.53)	0		0.00 (0.00)	35		0.45 (0.57)	19		0.25 (0.44)	30		0.31 (0.55)	19		0.38 (0.53)
	Self_PROM	77	0.25 (0.52)	0		0.00 (0.00)	28		0.36 (0.60)	16		0.21 (0.47)	19		0.20 (0.45)	14		0.28 (0.57)
**PREM^c^**																		
	PREM (total)	234	0.77 (0.92)	4		1.00 (0.00)	66		0.85 (0.93)	69		0.92 (1.06)	69		0.72 (0.85)	25		0.50 (0.79)
	Treatment	29	0.10 (0.32)	0		0.00 (0.00)	10		0.13 (0.37)	9		0.12 (0.37)	7		0.07 (0.26)	3		0.06 (0.24)
	Technology	23	0.08 (0.31)	1		0.25 (0.50)	7		0.09 (0.30)	1		0.01 (0.12)	7		0.07 (0.26)	6		0.12 (0.52)
	Satisfaction	98	0.32 (0.53)	2		0.50 (0.58)	26		0.33 (0.55)	35		0.47 (0.60)	26		0.27 (0.49)	9		0.18 (0.39)
	Self_PREM	84	0.28 (0.59)	1		0.25 (0.50)	23		0.29 (0.58)	24		0.32 (0.64)	29		0.30 (0.63)	7		0.14 (0.4)

^a^PROM: patient-reported outcome measure.

^b^HRQoL: health-related quality of life.

^c^PREM: patient-reported experience measure.

### Subgroup Analysis: Study Type

The second subgroup analysis was conducted based on the study type to evaluate the use frequency of PROMs and PREMs in different types of studies and the levels of evidence they were associated with. Of the 303 studies, 67 (22.1%) feasibility studies, 70 (23.1%) noncontrolled trials, 20 (6.6%) controlled trials, and 146 (48.2%) RCTs were identified. The study design served as an indicator of the evidence level of the studies [5]. The evidence level was determined according to the guidelines of the Oxford Centre for Evidence-based Medicine [47]. Study types with evidence level 3, such as feasibility studies (mean 1.66, SD 1.66) and noncontrolled trials (mean 1.66, SD 1.64), used fewer PROMs than controlled trials (mean 2.65, SD 2.72), with evidence level 2 or even RCTs (mean 4.12, SD 2.36), with evidence level 1. An opposite trend was observed for PREMs. The values for PREMs in order of increasing evidence level were as follows: feasibility study (mean 1.22, SD 0.87), noncontrolled trial (mean 1.00, SD 1.14), controlled trial (mean 0.70, SD 0.86), and RCT (mean 0.46, SD 0.71). The correlation analysis for the relationship between the number of PROMs or PREMs and the evidence levels resulted in r=−0.50 for PROMs and r=0.34 for PREMs (Figure 3). Table 6 lists the complete distribution of outcomes by study type.

**Table 6:** Outcomes by study type.

Outcomes		All (n=301)		Feasibility study (n=67)		Noncontrolled trial (n=70)		Controlled trial (n=20)		Randomized controlled trial (n=146)	
		∑	Mean value (SD)	∑	Mean value (SD)	∑	Mean value (SD)	∑	Mean value (SD)	∑	Mean value (SD)
**PROM^a^**											
	PROM	884	2.91 (2.40)	111	1.66 (1.66)	116	1.66 (1.64)	53	2.65 (2.72)	601	4.12 (2.36)
	HRQoL^b^	310	1.02 (1.05)	33	0.49 (0.79)	38	0.54 (0.79)	22	1.10 (0.91)	217	1.49 (1.07)
	Physical function	57	0.19 (0.45)	3	0.04 (0.21)	4	0.30 (0.23)	3	0.15 (0.49)	47	0.32 (0.56)
	Social function	9	0.03 (0.17)	0	0 (0)	2	0.03 (0.17)	0	0 (0)	7	0.05 (0.21)
	Emotional function	244	0.80 (1.18)	25	0.37 (0.69)	32	0.00 (0.90)	11	0.55 (1.00)	176	1.21 (1.36)
	Cognitive function	38	0.13 (0.40)	4	0.06 (0.24)	4	0.46 (0.23)	6	0.30 (0.57)	24	0.16 (0.57)
	Health literacy	37	0.12 (0.38)	8	0.12 (0.41)	5	0.06 (0.26)	3	0.15 (0.49)	19	0.13 (0.38)
	Side effects	8	0.03 (0.18)	3	0.04 (0.27)	1	0.07 (0.12)	0	0 (0)	4	0.03 (0.16)
	Adherence	103	0.34 (0.53)	21	0.31 (0.53)	15	0.01 (0.41)	3	0.15 (0.37)	64	0.44 (0.58)
	Self_PROM	77	0.25 (0.52)	14	0.21 (0.41)	15	0.21 (0.45)	5	0.25 (0.44)	43	0.29 (0.60)
**PREM^c^**											
	PREM	234	0.77 (0.92)	82	1.22 (0.87)	70	1.00 (1.44)	14	0.70 (0.86)	67	0.46 (0.71)
	Treatment	29	0.10 (0.32)	4	0.06 (0.24)	3	0.04 (0.20)	1	0.05 (0.22)	21	0.14 (0.39)
	Technology	23	0.08 (0.31)	12	0.18 (0.49)	3	0.04 (0.20)	0	0 (0)	7	0.05 (0.24)
	Satisfaction	98	0.32 (0.53)	32	0.48 (0.59)	34	0.49 (0.63)	7	0.35 (0.59)	25	0.17 (0.38)
	Self_PREM	84	0.28 (0.59)	34	0.51 (0.68)	30	0.43 (0.77)	6	0.30 (0.57)	14	0.10 (0.34)

^a^PROM: patient-reported outcome measure.

^b^HRQoL: health-related quality of life.

^c^PREM: patient-reported experience measure.

### Use Cases

Three use cases were formed to check the results for transferability and were based on common combinations of medical purpose and application type. The use cases were telemonitoring for cancer diseases (21/303, 6.9%), teleconsultation for mental and behavioral disorders (22/303, 7.3%), and telerehabilitation for cardiovascular diseases (21/303, 6.9%). Although the total number of studies on telemonitoring for diseases of the circulatory system was 22, we chose to cover the widest possible range of characteristics within the presented use cases. Therefore, we opted for telemonitoring for cancer diseases and telerehabilitation for cardiovascular diseases, although these have lower numbers.

**Figure 9:**
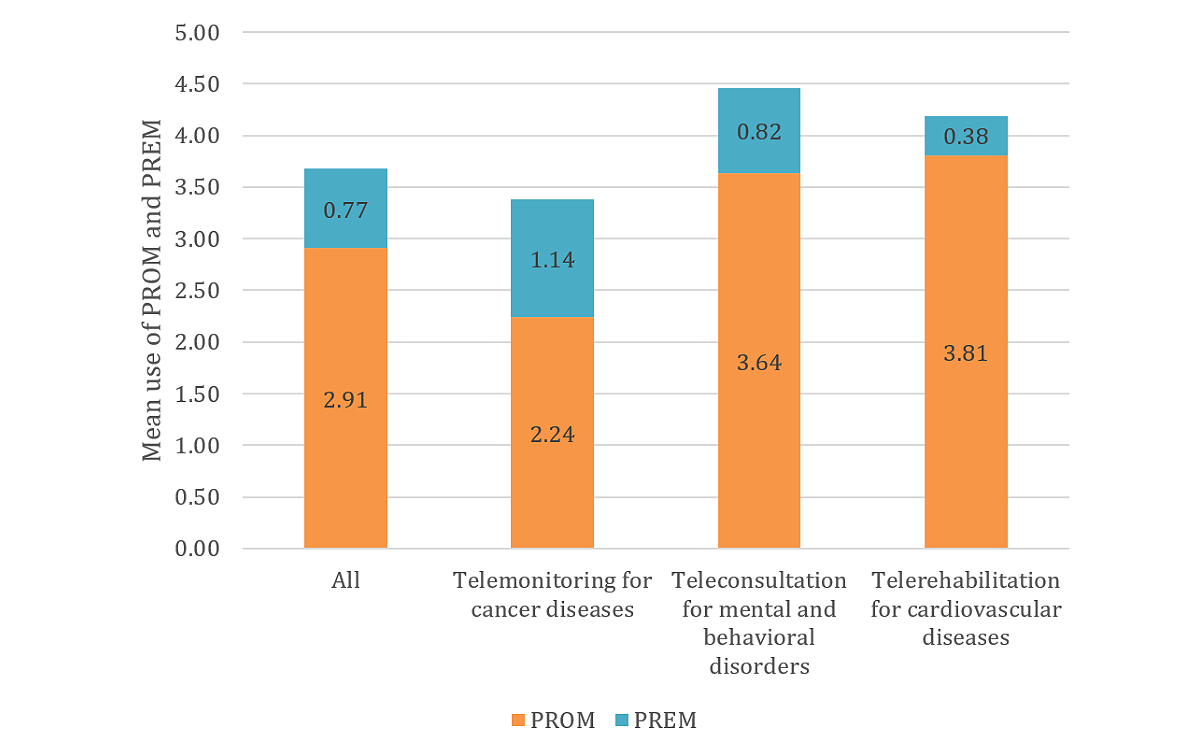
Use of patient-reported outcome measures and patient-reported experience measures by use cases. PREM: patient-reported experience measure; PROM: patient-reported outcome measure.

A descriptive analysis of the distribution of PROMs and PREMs and their outcome domains was also conducted. Again, the ratio of PROMs was different from that of PREMs (Figure 9). Similarly, the proportion of PREMs in the use case of telemonitoring for cancer diseases with evidence level 3 was higher than in the other 2 use cases with evidence level 1. HRQoL and emotional function were found to be the most frequently used outcome domains in all 3 cases (Table 7). Only the third most frequent outcome, satisfaction, was case-specific; it accounted for half of the cases. The results of the entire sample could be transferred to the 3 use cases, which could be an indication of the transferability of the review results to specific use cases.

**Table 7:** Use cases.

Characteristics			Use cases						
			All studies (n=303)		Telemonitoring for cancer diseases (n=21)		Teleconsultation for mental and behavioral disorders (n=22)		Telerehabilitation for cardiovascular diseases (n=21)
**Most common outcomes**									
	1	HRQoL^a^		HRQoL		Emotional function		HRQoL	
	2	Emotional function		Emotional function		HRQoL		Emotional function	
	3	Adherence		Satisfaction		Satisfaction and adherence		Physical function	
**Evidence level**									
	Modus	1		3		1		1	

^a^HRQoL: health-related quality of life.

## Discussion

### Summary and Discussion of Main Findings

The aim of this systematic review was to empirically examine the characteristics of PROM and PREM use in evaluation studies of telemedicine applications. Owing to the large number of possible combinations of application types (n=6) and medical purposes (n=24), there was great heterogeneity in the evaluation studies. Of the 144 possible combinations, 51 (35.4%) were identified in this study. However, we were able to answer the research questions.

PROMs dominated the evaluation of telemedicine applications. In total, 80% (4/5) of OMIs were PROMs, and only in 14% (1/7) of studies was no PROM used. On the other hand, PREMs were used in less than half of the studies, and hardly any of these PREMs were adapted to telemedical care. The lack of telemedicine-specific OMIs was apparently compensated for by the use of self-developed OMIs. This could indicate that the existing OMIs could not be applied because of the great heterogeneity of the telemedicine-specific use cases, did not collect the desired outcomes, or were simply not known to the evaluation team. The review by Hajesmaeel-Gohari and Bahaadinbeigy [48] in 2021 examined the use of validated telemedicine-specific OMIs in the form of PREMs for the evaluation of telemedicine service quality. The review was able to identify 59 different PREMs, of which only the 10 most frequent were mentioned. Our review was able to identify 70% (7/10) of the most frequent PREMs. However, the frequency distributions of the OMIs used do not match between the two reviews, as Hajesmaeel-Gohari and Bahaadinbeigy [48] identified a higher number of PREMs because of a more specific search strategy. They concluded that the use of PREMs for the evaluation of the quality of telemedicine applications should be obligatory and needs to be expanded, which also requires the development of further specific OMIs [48].

The quantity of PREMs decreased with an increasing number of PROMs; that is, a negative correlation (r=−0.23) was observed. One explanation for this correlation could be that the number of OMIs and outcome domains was kept as low as possible. In the sample, the median was 3 OMIs and outcome domains per study. However, the number of outcome domains per study varied (SD 2.36). As the OMIs are constructs of several items, depending on the instrument, this can range from a handful to several dozen items; the total number of items should be taken into account when selecting the OMIs [48]. Furthermore, the study participants or patients should not be overwhelmed by the total number of OMIs and included items as this could lead to incomplete answers or even dropout [49].

The number of telemedicine studies that collected PROMs and PREMs increased on average over time (Figures 5 and 6). In addition, the proportion of high-evidence studies, especially RCTs, also increased (Figure 6). It was shown that in years with a high proportion of high-evidence studies, the ratio of PROMs was considerably higher than the ratio of PREMs, as described above. This could be caused by the wider recognition and implementation of PROMs and PREMs [50,51], as can be seen in Figure 7, where the growth rate of studies using PROMs and PREMs is far higher than the growth rate of telemedicine papers in MEDLINE. The trend toward the increased use of PROMs and PREMs is also evident in several medical disciplines, such as oncology [52] and orthopedics [53], as well as in studies for regulatory purposes for medical devices [54].

In addition, guidelines that recommend the use of PROMs and PREMs published in recent years (eg, MAST 2012 [2] and NICE framework 2019 [3]) could have promoted the increased use of PROMs and PREMs over the years. These guidelines also recommend the use of high-evidence study designs. Again, an increased use of RCTs has been noticed since the publication of these guidelines.

Regardless of the telemedicine evaluation tools used, variations can be found between countries regarding the state of PROM and PREM implementation, types of data use, conditions and therapeutic areas, and challenges and success factors for PREM and PROM use [55]. Hence, regional and cultural aspects must be taken into account when developing, translating, and implementing PROMs, especially if they are measured using electronic tools [56]. Furthermore, these aspects have to be considered when evaluating PROM and PREM scores and comparing them between different countries.

The ratio of PROMs to PREMs also depended on the study type and evidence level. Although in low-evidence studies the frequency of PREMs was almost equal to the frequency of PROMs, it decreased with increasing evidence level. At the same time, more outcomes were recorded at high evidence levels (Figure 6). This could be related to the development cycle of telemedicine technologies [5]. Using evidence level as a surrogate parameter for the maturity stage of the application, feasibility studies and proof-of-concept studies increasingly require information on the usability and acceptance of the technology in addition to the clinical effectiveness. On the other hand, PREMs played almost no role in clinical trials with high evidence levels. PROMs clearly dominated in RCTs in relative and absolute numbers. This is also reflected in the Khoja-Durrani-Scott framework for eHealth evaluation [6]. Khoja et al [6] subdivided the development cycle of an eHealth application into 4 phases. The framework recommends focusing on typical PREM domains, such as usability, user-friendliness, and acceptance in the early phases of development. In later phases, evaluation should focus on health outcomes, such as quality of life and health impact, although these should also be recorded in the early phases. The design and evaluation framework for digital health interventions by Kowatsch et al [5] goes one step further and specifies the outcomes as well as the required study designs for each phase. With each phase, the evidence level of the study designs increases, and the focus of the outcomes change according to the needs. The first phase, the preparation phase, includes feasibility and acceptability studies to determine the ease of use and adherence. In the optimization phase, the first evidence of effectiveness, expected benefits, and satisfaction with the quality of the application should be measured. In the later phases, that is, the evaluation and implementation phases, the success of the implementation of digital health applications should be monitored. The fact that the selection of the evaluation design and outcomes should be made according to the stage of development and should have an appropriate level of evidence has also been pointed out by the MAST model [2] and the evaluation principles of Arnold and Scheibe et al [4]. The correlation of the PREMs (τ=0.35) and the PROMs (τ=−0.45) with evidence level indicates that evaluation was performed as described in the guidelines for maturity stage–based evaluation.

A key milestone in the implementation of PROMs and PREMs in evaluation studies of telemedicine interventions was set by Germany in 2020 with the *Digital Care Act*. One significant innovation is that the costs for the use of so-called *digital health applications* will be reimbursed by statutory health insurance [7,57]. As a result, since October 2020, around 90% of the population is entitled to a wide range of mobile health applications in the areas of telerehabilitation, telemonitoring, and digital self-management [57]. Another significant innovation is that the assessment of bankability does not exclusively depend on the medical benefits, which, among clinical and epidemiological outcomes, could be assessed by PROMs, such as HRQoL, but also on the so-called patient-relevant improvement of structure and processes, which are mainly assessed by PROMs and PREMs. Examples of patient-relevant improvement of structure and processes are coping with difficulties in everyday life because of illness, facilitating access to care, health literacy, patient autonomy, reduction of therapy-related expenses, and burdens for patients and their relatives [7]. Medical benefits and patient-relevant improvements of structure and processes are now of equal importance in the approval process of digital health applications, and only one of the outcomes has to be more effective than standard care [7]. This represents a significant increase in the importance of PROMs and PREMs in evaluation studies of telehealth applications. The reason for including patient-relevant improvement of structure and processes as an outcome in evaluation studies was that digital health applications are considered to improve patient self-efficacy [58] and health-related behaviors, such as adherence [59] and health literacy [60]. In our review, 31.7% (96/303) of the included studies assessed the effects on adherence to medication or other therapies, and 10% (30/303) assessed health literacy. The Danish MAST does not mention the measurement of health-related behavior changes [2]. Within the NICE framework, originally developed in the United Kingdom, applications with the purpose of improving health-related behaviors are assigned to their own group [3]. However, neither the MAST nor the NICE framework explicitly recommends capturing adherence or health literacy for all types of applications. Health literacy is not only an outcome but it is also a critical precondition for the successful use of telemedicine by the patient in addition to digital literacy. To ensure the appropriate use of the technology and the assessment of PROMs and PREMs, proper training and guidance of the users is of at least equal relevance, according to the literature [56,61-64]. Therefore, health literacy should not only be included in the evaluation merely for reasons of measuring effectiveness; it is also a possible factor influencing purposeful and successful telemedicine use by the patients [58,60,65,66]. In summary, future developments will show to what extent and in which way innovations from Germany will affect the use of PROMs and PREMs in evaluation studies of telemedicine applications.

### Strengths and Limitations

One limitation of the study is that the medical purpose was classified by the ICD-10 chapters, all of which only describe a group of diseases and not the disease itself [20]. Chapter 1, for example, covers circulatory diseases, which include congenital heart defects, strokes, and aneurysms, all of which differ in etiology, symptoms, and therapy. There was a similar degree of heterogeneity in telemedicine applications. A more detailed distinction between user groups, setting, technical execution, and other criteria exists in the taxonomy used as a basis for the subgroup analysis, but this was not considered in our review [21]. The same applies to the analysis of single OMIs. The problem of heterogeneity is not an issue inherent only to this study. In their paper published in *Nature* in 2020, Guo et al [1] pointed out that the different types of interventions, medical purposes, and outcomes can lead to limitations in reviews of digital health interventions in general.

Another limitation was the large number of possible combinations of medical purpose, application, and study type. Nevertheless, several patterns were identified to answer the research questions, and the results of the entire sample could be transferred to use cases; thus, the influence of heterogeneity was not as great as initially assumed.

Another limitation might be that only 1 reviewer performed full-text screening. In the context of classical systematic reviews for the purpose of evidence synthesis of effectiveness or risk factors, screening by 2 reviewers is mandatory to minimize beta error. The approach of our review, on the other hand, was different. We intended to use the methodology of a systematic literature search to generate data for quantitative analysis. Owing to the 627 studies to be screened, an increased beta error in the form of missing studies seemed acceptable to us for reasons of research economics. As we wanted to conduct a plain descriptive analysis of the data with a total of 303 included studies, we did not consider the validity of the result to be compromised.

The strength of the review is that, to the best of our knowledge, this is the first systematic review investigating the characteristics of PROM and PREM use in evaluation studies of telemedicine applications covering all application types and medical purposes.

Reviews do exist for specific use cases; however, these usually do not cover all outcomes. Instead, they focus on selected outcomes for the purpose of evidence synthesis or do not focus exclusively on PROMs and PREMs [14-16,48,67-71].

Preliminary excerpts of the review results were presented to an expert audience of health care scientists at a conference in October 2020 [72].

### Implications for Future Research

High heterogeneity reflected by the multitude of OMIs used per outcome domain and a lack of standardization poses a challenge to the selection of PROMs [70,71] and PREMs. New developments and updated versions of existing guidelines for the evaluation of telemedicine could contribute to further standardization in the selection of outcome domains and OMIs [73].

The use case analysis indicated that the most common outcome domains were HRQoL and emotional function, which could be the first starting point for further efforts. Equally, user satisfaction and usability [48] as well as health literacy and adherence [7] should be taken into account, although these outcome domains were not frequently surveyed in our review.

Further investigation will be required to reveal how the use of PROMs and PREMs for the evaluation of telemedicine will evolve over the next few years and if the trends observed in this review will persist.

In addition, upcoming studies will have to investigate how a greater consideration of PROMs and PREMs in German approval and reimbursement procedures for digital health applications will affect the future use of PROMs and PREMs in evaluation studies of telemedicine applications.

### Conclusions

In recent years, there has been an increasing number of studies, particularly high-evidence studies, that use PROMs and PREMs to evaluate telemedicine services. Despite the great heterogeneity of telemedicine interventions and the associated evaluation approaches, several conclusions can be drawn. PROMs have been in the focus of evaluation studies. With the increasing maturity stage of telemedicine applications and higher evidence levels, the use of PROMs has increased. PREMs played a role, especially in the initial phases of application development, with low-evidence study designs. In this case, PREMs were primarily used to test the usability and acceptance of the application. Regardless of the findings, telemedicine-specific PREMs should be used more frequently and in a standardized manner to continuously evaluate telemedicine service quality, both during and after implementation.

The distribution of the outcome domains showed that only HRQoL and emotional function were assessed in almost all studies. Simultaneously, health literacy as a precondition for using the application adequately, alongside proper training and guidance, has rarely been reported. At the level of the OMIs, it was shown that many different OMIs were used for each domain. Further efforts should be pursued for the standardization of PROM and PREM collection in evaluation studies of telemedicine applications.

## Appendix

Multimedia Appendix 1 Search string.

Multimedia Appendix 2 Study list.

## Abbreviations

AMSTAR 2: A Measurement Tool to Assess systematic Reviews
HRQoL: health-related quality of life
ICD-10: International Statistical Classification of Diseases and Related Health Problems, 10th revision
MAST: Model for Assessment of Telemedicine
NICE: National Institute for Health and Care Excellence
OMI: outcome measurement instrument
PREM: patient-reported experience measure
PROM: patient-reported outcome measure
RCT: randomized controlled trial

## References

[1] Guo C, Ashrafian H, Ghafur S, Fontana G, Gardner C, Prime M (2020). Challenges for the evaluation of digital health solutions-A call for innovative evidence generation approaches. NPJ Digit Med.

[2] Kidholm K, Ekeland AG, Jensen LK, Rasmussen J, Pedersen CD, Bowes A, Flottorp SA, Bech M (2012). A model for assessment of telemedicine applications: MAST. Int J Technol Assess Health Care.

[3] National Institute for Health and Care Excellence (NICE) (2021). Evidence standards framework for digital health technologies.

[4] Arnold K, Scheibe M, Müller O, Schmitt J, und die CCS THOS Konsensgruppe (2016). [Principles for the evaluation of telemedicine applications: Results of a systematic review and consensus process]. Z Evid Fortbild Qual Gesundhwes.

[5] Kowatsch T, Otto L, Harpernink S, Cotti A, Schlieter H (2019). A design and evaluation framework for digital health interventions. it - Inf Technol.

[6] Khoja S, Durrani H, Scott RE, Sajwani A, Piryani U (2013). Conceptual framework for development of comprehensive e-health evaluation tool. Telemed J E Health.

[7] Federal Institute for Drugs and Medical Devices (BfArM) (2020). The fast-track process for digital health applications (DiGA) according to section 139e SGBV.

[8] Kidholm K, Clemensen J, Caffery LJ, Smith AC (2017). The model for assessment of telemedicine (MAST): a scoping review of empirical studies. J Telemed Telecare.

[9] Nwe K, Larsen ME, Nelissen N, Wong DC (2020). Medical mobile app classification using the national institute for health and care excellence evidence standards framework for digital health technologies: interrater reliability study. J Med Internet Res.

[10] Locke HN, Brooks J, Arendsen LJ, Jacob NK, Casson A, Jones AK, Sivan M (2020). Acceptability and usability of smartphone-based brainwave entrainment technology used by individuals with chronic pain in a home setting. Br J Pain.

[11] FDA-NIH Biomarker Working Group (2020). BEST (Biomarkers, EndpointS, and other Tools).

[12] Higgins J, Thomas J, Chandler J, Cumpston M, Li T, Page M, Welch VA (2019). Cochrane Handbook for Systematic Reviews of Interventions, Second edition.

[13] Bull C, Byrnes J, Hettiarachchi R, Downes M (2019). A systematic review of the validity and reliability of patient-reported experience measures. Health Serv Res.

[14] Jackson BD, Gray K, Knowles SR, De Cruz P (2016). EHealth technologies in inflammatory bowel disease: a systematic review. J Crohns Colitis.

[15] Lancaster K, Abuzour A, Khaira M, Mathers A, Chan A, Bui V, Lok A, Thabane L, Dolovich L (2018). The use and effects of electronic health tools for patient self-monitoring and reporting of outcomes following medication use: systematic review. J Med Internet Res.

[16] Bright P, Hambly K (2018). What is the proportion of studies reporting patient and practitioner satisfaction with software support tools used in the management of knee pain and is this related to sample size, effect size, and journal impact factor?. Telemed J E Health.

[17] Mackintosh A, Comabella C, Hadi M, Gibbons E, Roberts N, Fitzpatrick R (2010). PROM group construct and instrument type filters. Department of Public Health, University of Oxford, Oxford.

[18] Daliya P, Gemmill EH, Lobo DN, Parsons SL (2019). A systematic review of patient reported outcome measures (PROMs) and quality of life reporting in patients undergoing laparoscopic cholecystectomy. Hepatobiliary Surg Nutr.

[19] Aiyegbusi OL, Kyte D, Cockwell P, Marshall T, Gheorghe A, Keeley T, Slade A, Calvert M (2017). Measurement properties of patient-reported outcome measures (PROMs) used in adult patients with chronic kidney disease: a systematic review. PLoS One.

[20] World Health Organization (2011). ICD-10: International Statistical Classification of Diseases and Related Health Problems: Tenth Revision.

[21] Harst L, Timpel P, Otto L, Richter P, Wollschlaeger B, Lantsch H (2019). An empirically derived taxonomy of telemedicine - development of a standardized codebook. Proceedings of the 18th Deutscher Kongress für Versorgungsforschung.

[22] Harst L, Otto L, Timpel P, Richter P, Lantzsch H, Wollschlaeger B, Winkler K, Schlieter H (2021). An empirically sound telemedicine taxonomy – applying the CAFE methodology. J Public Health (Berl.).

[23] Fitch C (2004). Information systems in healthcare: mind the gap. Proceedings of the 37th Annual Hawaii International Conference on System Sciences.

[24] Dierks C (2001). Rechtliche und praktische probleme der integration von telemedizin — ein Problemaufriss. Rechtsfragen der Telemedizin.

[25] Fong B, Fong A, Li C (2020). Information technologies in medicine and digital health. Telemedicine Technologies: Information Technologies in Medicine and Digital Health.

[26] Bashshur R, Shannon G, Krupinski E, Grigsby J (2011). The taxonomy of telemedicine. Telemed J E Health.

[27] Schulz EG, Stahmann A, Neumann CL (2015). Telemedicine: interventional decentralised blood pressure telemonitoring (idTBPM). Swiss Med Wkly.

[28] Rogante M, Grigioni M, Cordella D, Giacomozzi C (2010). Ten years of telerehabilitation: a literature overview of technologies and clinical applications. NeuroRehabilitation.

[29] Fitzner K, Moss G (2013). Telehealth--an effective delivery method for diabetes self-management education?. Popul Health Manag.

[30] Sheridan N, Kenealy T, Kuluski K, McKillop A, Parsons J, Wong-Cornall C (2017). Are patient and carer experiences mirrored in the practice reviews of self-management support (Prisms) provider taxonomy?. Int J Integr Care.

[31] Piga M, Cangemi I, Mathieu A, Cauli A (2017). Telemedicine for patients with rheumatic diseases: systematic review and proposal for research agenda. Semin Arthritis Rheum.

[32] Timpel P, Oswald S, Schwarz PE, Harst L (2020). Mapping the evidence on the effectiveness of telemedicine interventions in diabetes, dyslipidemia, and hypertension: an umbrella review of systematic reviews and meta-analyses. J Med Internet Res.

[33] Dodd S, Clarke M, Becker L, Mavergames C, Fish R, Williamson PR (2018). A taxonomy has been developed for outcomes in medical research to help improve knowledge discovery. J Clin Epidemiol.

[34] Shea BJ, Reeves BC, Wells G, Thuku M, Hamel C, Moran J, Moher D, Tugwell P, Welch V, Kristjansson E, Henry DA (2017). AMSTAR 2: a critical appraisal tool for systematic reviews that include randomised or non-randomised studies of healthcare interventions, or both. Br Med J.

[35] EuroQol Group (1990). EuroQol - a new facility for the measurement of health-related quality of life. Health Policy.

[36] Ware JE, Sherbourne CD (1992). The MOS 36-item short-form health survey (SF-36). I. Conceptual framework and item selection. Med Care.

[37] Kroenke K, Spitzer RL, Williams JB (2001). The PHQ-9: validity of a brief depression severity measure. J Gen Intern Med.

[38] Larsen DL, Attkisson CC, Hargreaves WA, Nguyen TD (1979). Assessment of client/patient satisfaction: development of a general scale. Eval Program Plann.

[39] Broekhuis M, van Velsen L, Hermens H (2019). Assessing usability of eHealth technology: a comparison of usability benchmarking instruments. Int J Med Inform.

[40] Glasgow RE, Wagner EH, Schaefer J, Mahoney LD, Reid RJ, Greene SM (2005). Development and validation of the Patient Assessment of Chronic Illness Care (PACIC). Med Care.

[41] Gorst SA, Coates L (2014). Telehealth acceptance measure: to assess patient motivation in the use of telehealth. The University of Manchester, Manchester.

[42] Stoyanov SR, Hides L, Kavanagh DJ, Zelenko O, Tjondronegoro D, Mani M (2015). Mobile app rating scale: a new tool for assessing the quality of health mobile apps. JMIR Mhealth Uhealth.

[43] Agha Z, Schapira RM, Laud PW, McNutt G, Roter DL (2009). Patient satisfaction with physician-patient communication during telemedicine. Telemed J E Health.

[44] Hirani SP, Rixon L, Beynon M, Cartwright M, Cleanthous S, Selva A, Sanders C, Newman SP, WSD Investigators (2017). Quantifying beliefs regarding telehealth: development of the Whole Systems Demonstrator Service User Technology Acceptability Questionnaire. J Telemed Telecare.

[45] Demiris G, Speedie S, Finkelstein S (2000). A questionnaire for the assessment of patients' impressions of the risks and benefits of home telecare. J Telemed Telecare.

[46] Parmanto B, Lewis AN, Graham KM, Bertolet MH (2016). Development of the Telehealth Usability Questionnaire (TUQ). Int J Telerehabil.

[47] Phillips B, Ball C, Sackett D, Badenoch D, Straus S, Haynes B (2009). Oxford centre for evidence-based medicine: levels of evidence (March 2009). Centre for Evidence-Based Medicine, University of Oxford.

[48] Hajesmaeel-Gohari S, Bahaadinbeigy K (2021). The most used questionnaires for evaluating telemedicine services. BMC Med Inform Decis Mak.

[49] DeWalt DA, Rothrock N, Yount S, Stone AA, PROMIS Cooperative Group (2007). Evaluation of item candidates: the PROMIS qualitative item review. Med Care.

[50] Raine R, Fitzpatrick R, Barratt H, Bevan G, Black N, Boaden R, Bower P, Campbell M, Denis Jl, Devers K, Dixon-Woods M, Fallowfield L, Forder J, Foy R, Freemantle N, Fulop Nj, Gibbons E, Gillies C, Goulding L, Grieve R, Grimshaw J, Howarth E, Lilford Rj, McDonald R, Moore G, Moore L, Newhouse R, O’Cathain A, Or Z, Papoutsi C, Prady S, Rycroft-Malone J, Sekhon J, Turner S, Watson Si, Zwarenstein M (2016). Challenges, solutions and future directions in the evaluation of service innovations in health care and public health. Health Serv Deliv Res.

[51] Mercieca-Bebber R, King MT, Calvert MJ, Stockler MR, Friedlander M (2018). The importance of patient-reported outcomes in clinical trials and strategies for future optimization. Patient Relat Outcome Meas.

[52] Kargo AS, Coulter A, Jensen PT, Steffensen KD (2019). Proactive use of PROMs in ovarian cancer survivors: a systematic review. J Ovarian Res.

[53] Siljander MP, McQuivey KS, Fahs AM, Galasso LA, Serdahely KJ, Karadsheh MS (2018). Current trends in patient-reported outcome measures in total joint arthroplasty: a study of 4 major orthopaedic journals. J Arthroplasty.

[54] Weszl M, Rencz F, Brodszky V (2019). Is the trend of increasing use of patient-reported outcome measures in medical device studies the sign of shift towards value-based purchasing in Europe?. Eur J Health Econ.

[55] Steinbeck V, Ernst SC, Pross C (2021). Patient-Reported Outcome Measures (PROMs): ein internationaler Vergleich. Bertellsmann Stiftung, Gütersloh.

[56] Coons SJ, Eremenco S, Lundy JJ, O'Donohoe P, O'Gorman H, Malizia W (2015). Capturing Patient-Reported Outcome (PRO) data electronically: the past, present, and promise of ePRO measurement in clinical trials. Patient.

[57] Gerke S, Stern AD, Minssen T (2020). Germany's digital health reforms in the COVID-19 era: lessons and opportunities for other countries. NPJ Digit Med.

[58] Conard S (2019). Best practices in digital health literacy. Int J Cardiol.

[59] Hamine S, Gerth-Guyette E, Faulx D, Green BB, Ginsburg AS (2015). Impact of mHealth chronic disease management on treatment adherence and patient outcomes: a systematic review. J Med Internet Res.

[60] Kim H, Xie B (2017). Health literacy in the eHealth era: a systematic review of the literature. Patient Educ Couns.

[61] Ly JJ, Crescioni M, Eremenco S, Bodart S, Donoso M, Butler AJ, Dallabrida SM (2019). Training on the use of technology to collect patient-reported outcome data electronically in clinical trials: best practice recommendations from the ePRO consortium. Ther Innov Regul Sci.

[62] Fleming S, Barsdorf AI, Howry C, O'Gorman H, Coons SJ (2015). Optimizing electronic capture of clinical outcome assessment data in clinical trials: the case of patient-reported endpoints. Ther Innov Regul Sci.

[63] Scheibe M, Reichelt J, Bellmann M, Kirch W (2015). Acceptance factors of mobile apps for diabetes by patients aged 50 or older: a qualitative study. Med 2 0.

[64] Scheibe M, Lang C, Druschke D, Arnold K, Luntz E, Schmitt J, Holthoff-Detto V (2021). Independent use of a home-based telemonitoring app by older patients with multimorbidity and mild cognitive impairment: qualitative study. JMIR Hum Factors.

[65] Smith B, Magnani JW (2019). New technologies, new disparities: the intersection of electronic health and digital health literacy. Int J Cardiol.

[66] Estacio EV, Whittle R, Protheroe J (2019). The digital divide: examining socio-demographic factors associated with health literacy, access and use of internet to seek health information. J Health Psychol.

[67] Farzandipour M, Nabovati E, Sharif R, Arani MH, Anvari S (2017). Patient self-management of asthma using mobile health applications: a systematic review of the functionalities and effects. Appl Clin Inform.

[68] Sul A, Lyu D, Park D (2020). Effectiveness of telemonitoring versus usual care for chronic obstructive pulmonary disease: a systematic review and meta-analysis. J Telemed Telecare.

[69] Yun JE, Park J, Park H, Lee H, Park D (2018). Comparative effectiveness of telemonitoring versus usual care for heart failure: a systematic review and meta-analysis. J Card Fail.

[70] Warrington L, Absolom K, Conner M, Kellar I, Clayton B, Ayres M, Velikova G (2019). Electronic systems for patients to report and manage side effects of cancer treatment: systematic review. J Med Internet Res.

[71] Doshi H, Hsia B, Shahani J, Mowrey W, Jariwala SP (2021). Impact of technology-based interventions on patient-reported outcomes in asthma: a systematic review. J Allergy Clin Immunol Pract.

[72] Knapp A (2020). Anwendung von PROMs und PREMs bei der evaluation von telemedizinischen anwendungen: überblick über die aktuelle praxis. Proceedings of the 19th Deutscher Kongress für Versorgungsforschung.

[73] Schlieter H, Timpel P, Otto L, Richter P, Wollschlaeger B, Knapp A, Harst L (2021). Digitale Gesundheitsanwendungen – Forderungen für deren entwicklung, implementierung und begleitende evaluation. Monitor Versorgungsforschung.

