# Supplementary material for: Use of Patient-Reported Outcome Measures and Patient-Reported Experience Measures Within Evaluation Studies of Telemedicine Applications: Systematic Review
Source: J Med Internet Res. 2021 Nov 17;23(11):e30042. doi: 10.2196/30042 (PMC8663685; doi:10.2196/30042)
Supplement: Multimedia Appendix 1 [file jmir_v23i11e30042_app1.docx]

| **Number** | **MEDLINE (2.04.2020)** |  | **EMBASE (2.04.2020)** |
| --- | --- | --- | --- |
|  |  |  |  |
| 1 | telehealth or (telehealth or tele-health).ti,ab. | 1 | exp telehealth/ or (telehealth or tele-health).ti,ab. |
| 2 | telemedicine[Mesh] or (telemedicine or tele-medicine).ti,ab. | 2 | exp telemedicine/ or (telemedicine or tele-medicine).ti,ab. |
| 3 | (ehealth or e-health or "e health").ti,ab. | 3 | (ehealth or e-health or "e health").ti,ab. |
| 4 | ("mobile health" or m-health or mhealth or "m health").ti,ab. | 4 | ("mobile health" or m-health or mhealth or "m health").ti,ab. |
| 5 | "digital health".ti,ab. | 5 | "digital health".ti,ab. |
| 6 | (telecar* or tele-car*).ti,ab. | 6 | (telecar* or tele-car*).ti,ab. |
| 7 | (emedic* or e-medic* or "e medic*").ti,ab. | 7 | (emedic* or e-medic* or "e medic*").ti,ab. |
| 8 | teleconsultation [MeSH] or (teleconsultation or tele-consultation or tele-consil* or tele-consil*).ti,ab. | 8 | exp teleconsultation/ or (teleconsultation or tele-consultation or tele-consil* or tele-consil*).ti,ab. |
| 9 | telecounsel*or tele-counsel*.ti,ab. | 9 | telecounsel*or tele-counsel*.ti,ab. |
| 10 | (telediagnos* or tele-diagnos*).ti,ab. | 10 | exp telediagnosis/ or (telediagnos* or tele-diagnos*).ti,ab. |
| 11 | (teleambulance or tele-ambulance).ti,ab. | 11 | (teleambulance or tele-ambulance).ti,ab. |
| 12 | (telemonitor* or tele-monitor*).ti,ab. | 13 | (telemonitor* or tele-monitor*).ti,ab. |
| 13 | telerehabilitation [MeSH] or (telerehabilitation or tele-rehabilitation).ti,ab. | 14 | exp telerehabilitation/ or (telerehabilitation or tele-rehabilitation).ti,ab. |
| 14 | "remote rehabilitation"[MeSH] or "remote rehabilitation".ti,ab. | 14 | exp "remote rehabilitation"/ or "remote rehabilitation".ti,ab. |
| 15 | telehome [MeSH] or (telehome or tele-home).ti,ab. | 15 | (telehome or tele-home).ti,ab. |
| 16 | (telemanagement or tele-management).ti,ab. | 16 | (telemanagement or tele-management).ti,ab. |
| 17 | telenursing [MeSH] or (telenurs* or tele-nurs*).ti,ab. | 17 | exp telenursing/ or (telenurs* or tele-nurs*).ti,ab. |
| 18 | (telesupport or tele-support).ti,ab. | 18 | (telesupport or tele-support).ti,ab. |
| 19 | (teletherap* or tele-therap*).ti,ab. | 19 | exp teletherapy/ or (teletherap* or tele-therap*).ti,ab. |
| 20 | (telecardiolog* or tele-cardiolog*).ti,ab. | 20 | exp telecardiology/ or (telecardiolog* or tele-cardiolog*).ti,ab. |
| 21 | (telestroke or tele-stroke).ti,ab. | 21 | (telestroke or tele-stroke).ti,ab. |
| 22 | (teledermatol* or tele-dermatology).ti,ab. | 22 | exp teledermatology/ or (teledermatol* or tele-dermatology).ti,ab. |
| 23 | 23 teleradiology [MeSH] or (teleradiolog* or tele-radiolog*).ti,ab. | 23 | 23 exp teleradiology/ or (teleradiolog* or tele-radiolog*).ti,ab. |
| 24 | 1 or 2 or 3 or 4 or 5 or 6 or 7 or 8 or 9 or 10 or 11 or 12 or 13 or 14 or 15 or 16 or 17 or 18 or 19 or 20 or 21 or 22 or 23 | 24 | 1 or 2 or 3 or 4 or 5 or 6 or 7 or 8 or 9 or 10 or 11 or 12 or 13 or 14 or 15 or 16 or 17 or 18 or 19 or 20 or 21 or 22 or 23 |
| 25 | "patient reported outcome measures" [MeSH] or ("PROM" or "patient reported outcome measures" or "patientreported outcome measures" or "patient-reported outcome measures" or "patient reported outcom*" or "patientreported outcom*" or "patient-reported outcom*").ti,ab. | 25 | exp "patient reported outcome measures"/ or ("PROM" or "patient reported outcome measures" or "patientreported outcome measures" or "patient-reported outcome measures" or "patient reported outcom*" or "patientreported outcom*" or "patient-reported outcom*").ti,ab. |
| 26 | ("PREM" or "patient reported experience measures" or "patientreported experience measures" or "patient-reported experience measures" or "patient reported experience " or "patientreported experience " or "patient-reported experience ").ti,ab. | 26 | exp "patient reported experience measures"/ or ("PREM" or "patient reported experience measures" or "patientreported experience measures" or "patient-reported experience measures" or "patient reported experience " or "patientreported experience " or "patient-reported experience ").ti,ab. |
| 27 | (HR-PRO or HRPRO or HRQL or HRQoL or QL or QoL).ti,ab. or "quality of life".mp. or ("health index*" or "health indices" or "health profile*").ti,ab. or "health status".ti,ab. or ((patient or self or child or parent or carer or proxy) and (appraisal* or appraised or report or reported or reporting or rated or rating* or based or assessed or assessment*)).ti,ab. or ((disability or function or functional or functions or subjective or utility or utilities or wellbeing or well being) and (index or indices or instrument or instruments or measure or measures or questionnaire* or profile or profiles or scale or scales or score or scores or status or survey or surveys)).ti,ab. |  | (HR-PRO or HRPRO or HRQL or HRQoL or QL or QoL).ti,ab. or "quality of life".mp. or ("health index*" or "health indices" or "health profile*").ti,ab. or "health status".mp. or ((patient or self or child or parent or carer or proxy) adj (appraisal* or appraised or report or reported or reporting or rated or rating* or based or assessed or assessment*)).ti,ab. or ((disability or function or functional or functions or subjective or utility or utilities or wellbeing or well being) adj2 (index or indices or instrument or instruments or measure or measures or questionnaire* or profile or profiles or scale or scales or score or scores or status or survey or surveys)).ti,ab. |
| 28 | 25 or 26 or 27 | 28 | 25 or 26 or 27 |
| 29 | 24 and 28 | 29 | 24 and 28 |
| 30 | limit 29 to full text | 30 | limit 29 to full text |
| 31 | limit 30 to animals | 31 | limit 30 to animals |
| 32 | 30 not 31 | 32 | 30 not 31 |
| 33 | 32.af. not (letter or editorial or review or systematic review or meta-analysis).pt. | 33 | 32.af. not (letter or editorial or review or systematic review or meta-analysis).pt. |
|  |  |  |  |
| **Number of publications** |  |  |  |
|  | 1417 |  | 1254 |
|  |  |  |  |
| **Total** |  |  |  |
|  | 2671 |  |  |
